# Dual Access Rapid Trap for Augmentation of Guide Catheter Support in the Left Coronary System

**DOI:** 10.1016/j.jscai.2024.102240

**Published:** 2024-07-29

**Authors:** Adam Z. Spitz, Augustin J. Delago, Jason Wollmuth, Eric S. Rothstein

**Affiliations:** a Dartmouth-Hitchcock Medical Center Heart and Vascular Center, Geisel School of Medicine, Lebanon, New Hampshire; b Providence Heart and Vascular Center, Providence St. Vincent Medical Center, Portland, Oregon

**Keywords:** complex high-risk indicated procedure, percutaneous coronary intervention, stenting technique

## Abstract

Coaxial trapping of guide extension catheters within a coronary artery allows for maximal support in delivering equipment; however, use of this technique within the left main coronary artery has been avoided due to concerns surrounding hemodynamic instability and vessel injury. We describe our initial experience with impromptu coaxial trapping of guide extension catheters within the left main coronary artery using the dual access rapid trap (DART) technique. Coaxial trapping of guide extension catheters within the left main coronary artery using DART enabled successful equipment delivery across balloon-uncrossable lesions. This case demonstrates that DART is a safe and effective method for equipment delivery across balloon-uncrossable lesions and may aid operators in safely solving complex cases.

## Introduction

Appropriate guide catheter (GC) selection is paramount to performing successful percutaneous coronary intervention (PCI) as adequate support is necessary for delivery of equipment to the target lesion. In many cases it is necessary to increase support without changing catheters. Guide extension catheters (GECs) are commonly used to improve support, although they often do not provide enough support for delivery of gear through challenging anatomy or balloon-uncrossable lesions. Coaxial trapping of a GEC inside a coronary artery allows for maximizing support in order to deliver gear. This has been discouraged in the left main coronary artery (LMCA) due to concerns surrounding hemodynamic instability and vessel injury. We describe our initial experience with impromptu GEC trapping in the LMCA utilizing the dual access rapid trap (DART) technique.

## The DART technique

**Figure 1:**
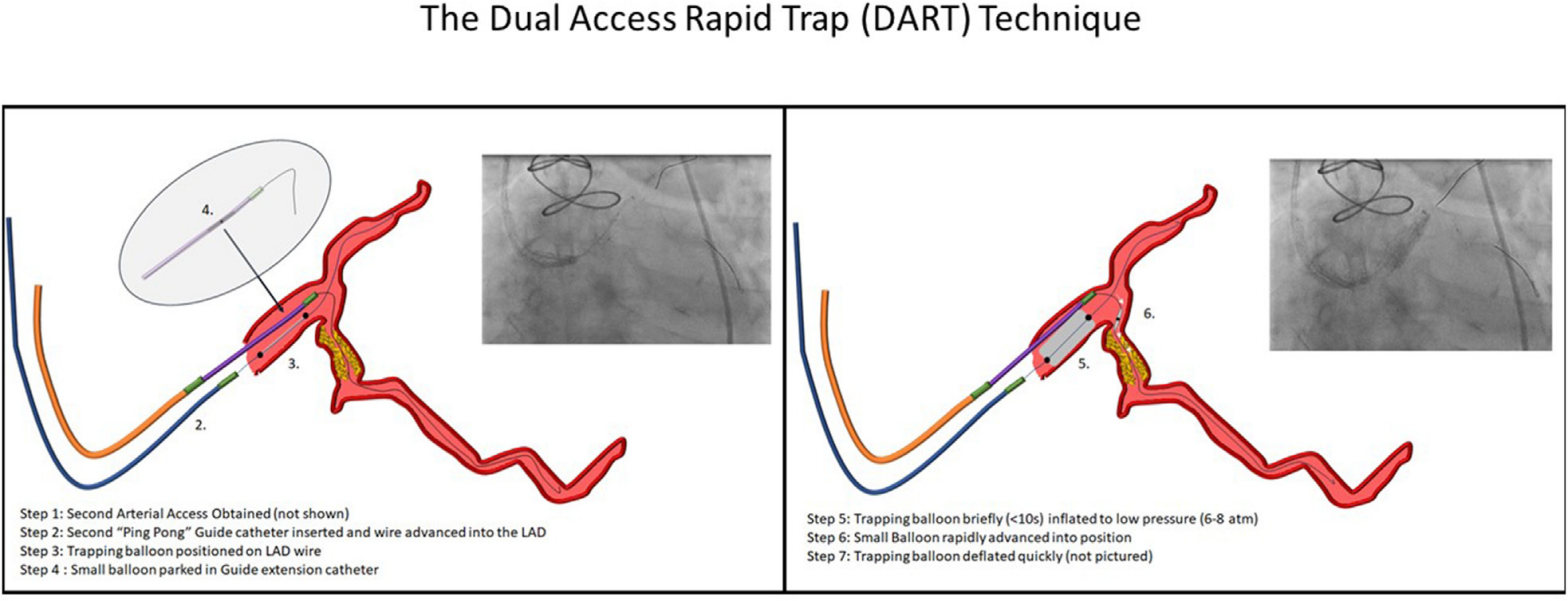
**Step-by-step illustration demonstrating how to perform DART.** DART, dual access rapid trap; LAD, left anterior descending artery.

A second (≥5F) arterial access point is obtained, and a second GC is advanced to the left coronary cusp. The initial GC is slightly withdrawn from the artery (leaving the GEC in the LMCA), and the second GC is positioned in the LMCA. A wire is advanced to any distal vessel, and a compliant balloon (sized at two-thirds the diameter of the LMCA) is advanced over this wire into the LMCA. The device needing to be delivered is advanced through the primary GC to the distal edge of the GEC. While coordinating as a team, the second operator rapidly inflates the balloon in the LMCA from the second GC to low pressure (5-7 atm), trapping the GEC from the initial system. The primary operator rapidly advances the equipment to the desired position and, once positioned, the second operator quickly deflates the LMCA trapping balloon within 5 to 10 seconds to prevent hemodynamic instability (Figure 1).

## Case

**Figure 2:**
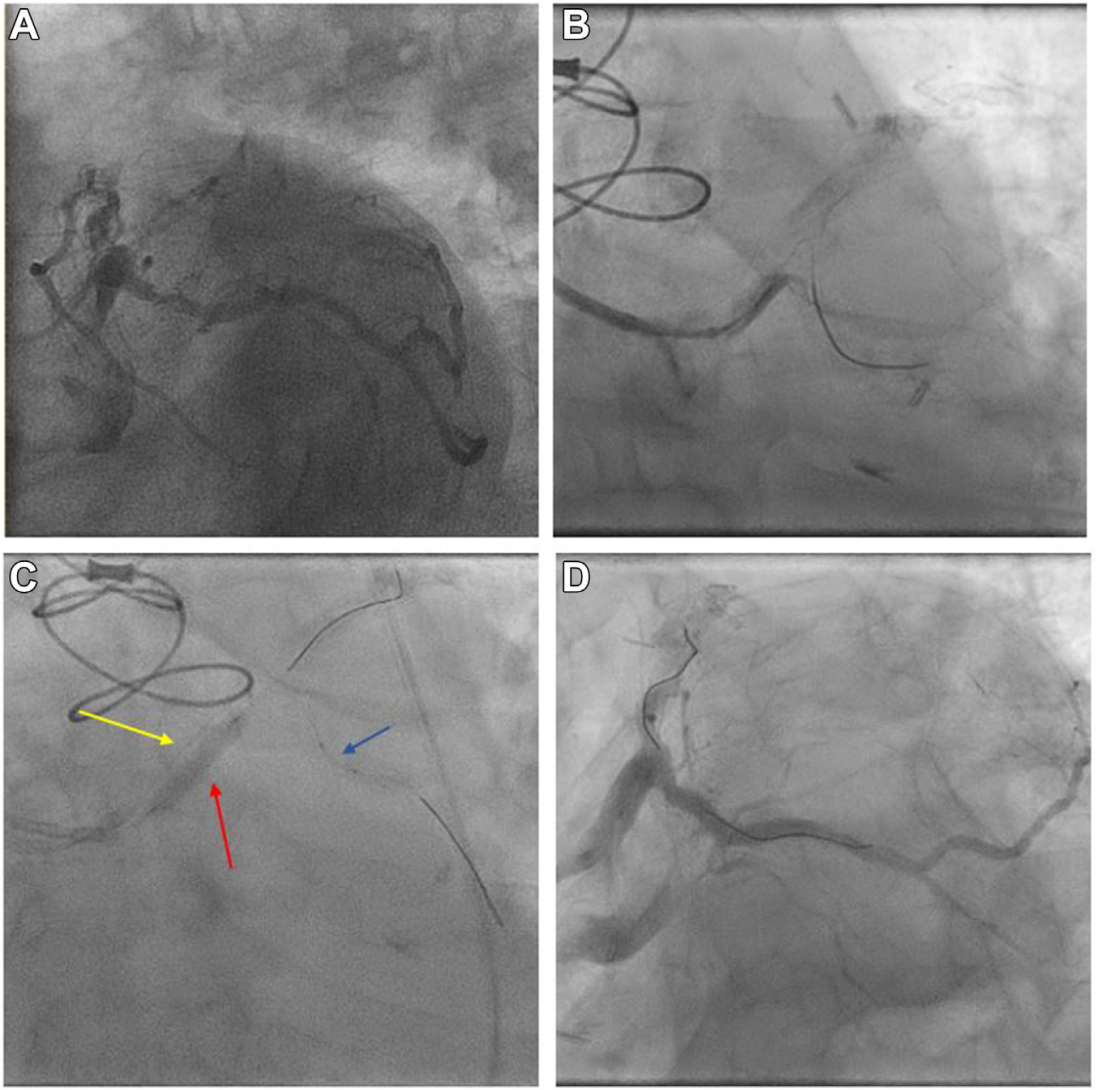
**DART Case 1.** (**A**) Subtotal, heavily calcified left circumflex artery lesion. (**B**) Attempts to wire the lesion resulting in wire prolapse due to inadequate support. (**C**) DART technique showing anchoring balloon (red arrow), anchored GEC (yellow arrow), and balloon successfully delivered to target lesion (blue arrow). (**D**) Final angiographic result. DART, dual access rapid trap; GEC, guide extension catheter.

A 76-year-old man with history of coronary artery bypass grafting presented with angina and inferolateral ischemia on stress testing. Angiography performed from the right radial approach demonstrated a subtotal, heavily calcified occlusion in a retroflexed left circumflex artery that was tented by an occluded saphenous vein graft (Figure 2A). Initial wiring of the vessel was unsuccessful due to lack of GC support (Figure 2B). Attempts to support wiring with a GEC, a low profile microcatheter, and an angled microcatheter were also unsuccessful. Second access was obtained and DART was performed (Figure 2C). This markedly improved support, which allowed for wiring of the lesion crossing with a Sion Blue wire (Asahi Intecc). Subsequent DART inflations allowed for delivery of low profile balloons, followed by noncompliant and Shockwave intravascular lithotripsy balloons (Shockwave Medical), and finally, a 3.5 × 18 mm drug-eluting stent. Final angiographic and intravascular ultrasound imaging demonstrated an optimized stent with no evidence of dissection in the LMCA (Figure 2D).

## Discussion

During complex PCI in calcified, tortuous, and totally occluded vessels, GC support is vital to procedural success. Traditional methods to augment support all have limitations, and challenging anatomy requires comfort with a wide variety of techniques. In that light, we present a case of successful complex PCI facilitated by the utilization of DART.

Elrayes et al^1^ previously described approaches to support escalation strategies in balloon-uncrossable lesions. In particular, the authors discuss the utilization of coaxial GEC anchoring to provide support, but that practice has been discouraged in an unprotected LMCA. Koutouzis et al^2^ demonstrated the “ping-pong” technique for treating a balloon-uncrossable chronic total occlusion (CTO), with support gained from an adjacent wire.

DART was inspired by the initial published experience utilizing coaxial GEC trapping in the right coronary artery for CTO-PCI^1^^,^^3^ and represents an evolution of this initial technique to allow for its use in an unprotected LMCA without hemodynamic or anatomic compromise. In the case described above, as well as in subsequent cases in which DART was utilitized, our patients experienced no angina or hypotension with brief occlusion of the LMCA, and the vessel was examined both angiographically and with intravascular ultrasound post-PCI, with no evidence of dissection. Although there are limitations to this technique, it allows for efficient augmentation of support without “giving up position” or dealing with issues related to equipment or GC incompatibility. Of note, in the case presented, the patient was hemodynamically stable, well compensated, and had normal left ventricular systolic function pre-PCI. Caution should be taken utilizing this technique in patients with diminished cardiac reserve or those who are unstable. Additionally, we have not utilized this technique for advancing retrograde gear during right coronary artery CTO-PCI, and caution should still be taken prior to occlusion of a last remaining coronary vessel. Given concerns for possible hemodynamic instability and left main dissection, operators should be comfortable and proficient with left main PCI and mechanical circulatory support prior to using this technique.

This is the first published case demonstrating the feasibility of a technique, DART, for rapid coaxial GEC trapping in an unprotected LMCA. This technique adds to the repertoire of potential solutions for balloon-uncrossable lesions, and we believe that this may aid other operators in safely solving complex cases.

## Conclusion

We present our initial experience utilizing DART in the LMCA, with DART allowing for equipment delivery across balloon-uncrossable lesions.
